# Editorial: Engineering *Corynebacterium glutamicum* Chassis for Synthetic Biology, Biomanufacturing, and Bioremediation

**DOI:** 10.3389/fbioe.2022.923145

**Published:** 2022-06-08

**Authors:** Yu Wang, Ping Zheng, Zhenghong Xu, Akihiko Kondo, Christoph Wittmann, Volker F. Wendisch

**Affiliations:** ^1^ Key Laboratory of Systems Microbial Biotechnology, Tianjin Institute of Industrial Biotechnology, Chinese Academy of Sciences, Tianjin, China; ^2^ The Key Laboratory of Industrial Biotechnology, Ministry of Education, School of Biotechnology, Jiangnan University, Wuxi, China; ^3^ Department of Chemical and Engineering, Graduate School of Engineering, Kobe University, Kobe, Japan; ^4^ Institute of Systems Biotechnology, Saarland University, Saarbrücken, Germany; ^5^ Genetics of Prokaryotes, Faculty of Biology and CeBiTec, Bielefeld University, Bielefeld, Germany

**Keywords:** *Corynebacterium glutamicum*, synthetic biology, biomanufacturing, biochemicals, omics technology, gene regulation

The Gram-positive soil bacterium *Corynebacterium glutamicum* was discovered about 60 years ago as an l-glutamate producer and has become a leading workhorse in industrial biotechnology. It is now used for the industrial production of over 6 million tons of amino acids (such as l-lysine and l-glutamate) per year and shows great potential for producing many more compounds ranging from alcohols and organic acids to plant secondary metabolites (Wolf et al., 2021). Several characteristics of *C. glutamicum* make it particularly interesting for industrial biotechnology, such as the GRAS (generally regarded as safe) status of its products, fast growth with relatively few nutrient requirements, and capability of utilizing sugars, sugar alcohols, organic acids, and aromatic compounds. With the development of CRISPR-based genome editing methods and synthetic and systems biology tools, the ability to understand and engineer the metabolism and regulation of *C. glutamicum* has been extensively enhanced (Liu et al., 2022). However, over 40% of genes of the type strain ATCC 13032 have not been experimentally characterized for their biological functions and the metabolic and regulatory mechanisms underlying the superior industrial performance of *C. glutamicum* have not been fully deciphered. Research on metabolic modeling, chassis engineering, multi-omics understanding, genome mining, etc., is expected to further realize the potential of this bacterium in biomanufacturing of chemicals and proteins and bioremediation of pollutants.

**FIGURE 1:**
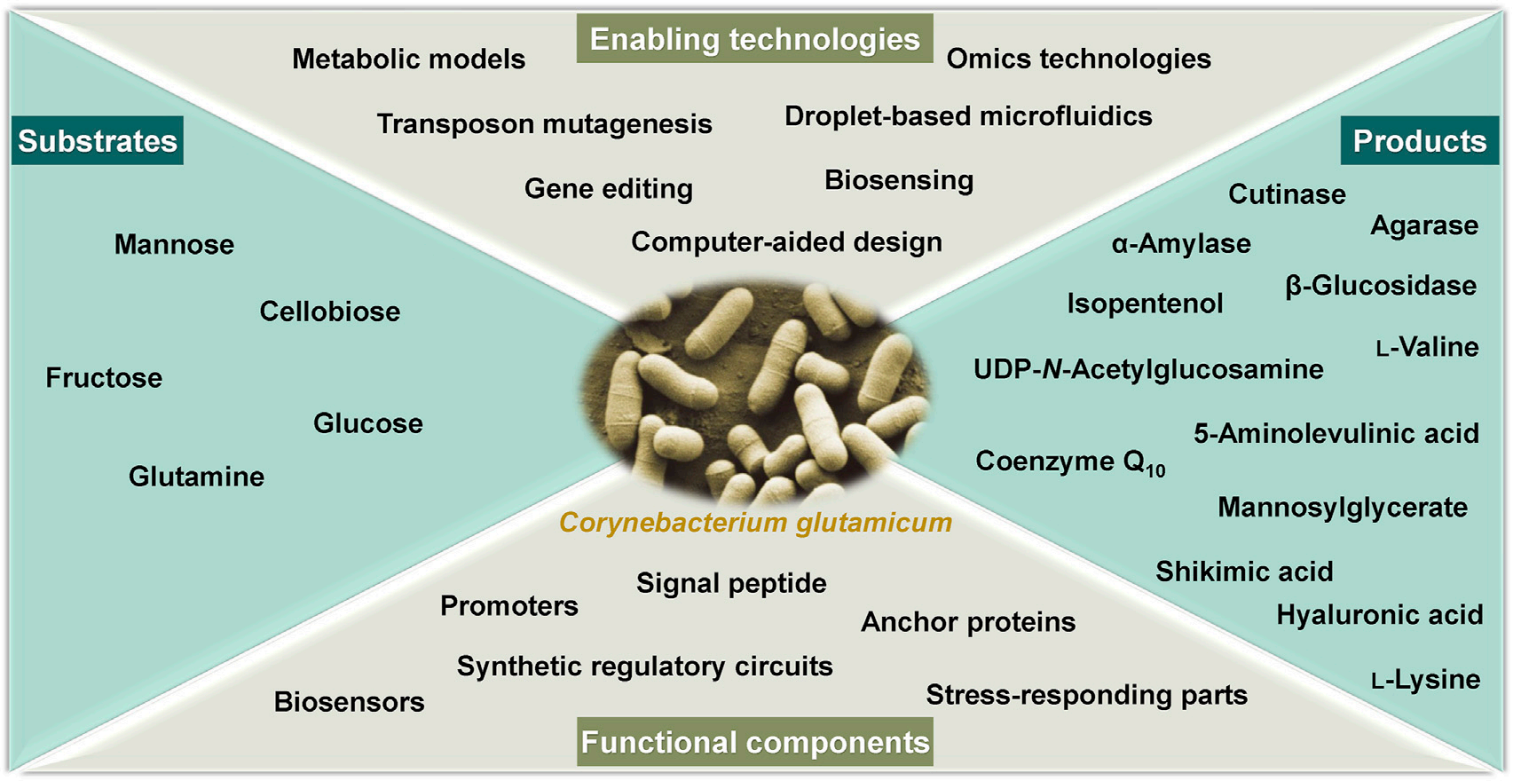
A general categorization of the keywords of the articles collected in this Research Topic.

A Research Topic of articles including 18 original research articles and 1 mini review article specialized in *C. glutamicum* research from the leading groups in this field is presented. Each article provides a state-of-the-art view of the metabolic engineering efforts, systems biology analyses, and/or technical advances. The Research Topic focuses on the development of enabling technologies, mining of functional components, and engineering of *C. glutamicum* as microbial cell factories for bioconversion of renewable feedstocks to useful chemicals and proteins (Figure 1).

It is great to see that a significant part of the Research Topic presents modern enabling technologies and their practical application in *C. glutamicum*. In the design-build-test-learn biological engineering cycle, genome-scale metabolic models (GEMs) are considered fundamental tools. Feierabend et al. presented an updated and unified GEM of *C. glutamicum* ATCC 13032 (iCGB21FR) with high quality regarding comprehensiveness and data standards. The *in silico* analysis may provide numerous designs for strain engineering. To bring these designs to life, computer-aided design tools and robotic system-assisted genome editing technologies will certainly help. Yang et al. developed a free online tool called GEDpm-cg for the design of genomic point mutations in *C. glutamicum*. Primers required for tool plasmid assembly and sequencing verification were quickly designed and provided, which would be useful for large-scale mutation analysis. As summarized by Wang et al., versatile genome editing tools including those based on the CRIPSR systems have been developed for *C. glutamicum*. Tailored design tools for not only point mutation but also large-scale genome engineering are still needed. In the test and learn parts, many high-throughput and systematic technologies including genome-scale transposon mutagenesis (Linder et al.), droplet-based microfluidics (Balasubramanian et al.), biosensing (Bakkes et al.), and multi-omics analyses (Kappelmann et al. and Banerjee et al.) have been used. Kappelmann et al. performed a comprehensive analysis of single or double deletion mutants in the anaplerosis of *C. glutamicum* under defined glucose conditions. Valuable information regarding the genotype-phenotype relationships in these mutants was unraveled by combining untargeted proteomics, quantitative metabolomics, and whole-genome sequencing. Banerjee et al. conducted genome and RNA sequencing of an engineered isopentenol-producing *C. glutamicum* strain under industrially relevant conditions including scale transition and ionic liquid stress. This omics information clarified the cell response of a *C. glutamicum* strain engineered to produce isopentenol.

The advances of enabling technologies considerably accelerate the running of the design-build-test-learn cycle and promote the engineering of *C. glutamicum* chassis, development of new components and circuits, and construction of microbial cell factories. To engineer a chassis that can efficiently metabolize fructose and channel the carbon flux to the oxidative pentose phosphate pathway for NADHP generation, Krahn et al. engineered and evolved fructose-utilizing mutants. Crucial mutations in the glucose phosphotransferase system enzymes were identified to explain the altered fructose uptake. A *C. glutamicum* chassis named CR101 with the removal of prophages and all insertion sequence (IS) elements was constructed by Linder et al. This chassis CR101 shows growth characteristics identical to the wild-type and increased transformability and could serve as an optimal host for basic research and biotechnology including genome-scale transposon mutagenesis.

Several articles collected in this Research Topic describe new functional components for engineering of *C. glutamicum*. To provide more available anchoring motifs for the display of recombinant proteins on the surfaces of *C. glutamicum* cells, Lin et al. predicted and screened 14 potential anchor proteins and identified 3 new anchoring proteins that performed better than the commonly used ones. Inducible gene expression systems are always important for reprogramming the metabolism and regulation of microorganisms. Three sets of gene regulation systems induced by myo-inositol, hyperosmotic stress, and phenolic compounds such as ferulic acid were developed and applied for dynamic control of gene expression. Lu et al. constructed a *myo*-inositol-inducible expression vector pMI-4 using the *iolR* ^*q*^ cassette and the *myo*-inositol-inducible promoter P_*iolT1*_, which was used for gene overexpression for 5-aminolevulinic acid production. Huang et al. characterized the promoters controlled by the two-component signal transduction system MtrA/MtrB responding to hyperosmotic stress. The promoter of *NCgl1418* that exhibited a high inducibility was further engineered and used to develop a CRISRPi system induced by hyperosmotic stress. Siebert et al. utilized the VanR/P_vanABK_^*^ regulatory system responding to phenolic compounds ferulic acid, vanillin, and vanillic acid to develop a timed off-switch for dynamic control of gene expression. With the depletion of exogenous phenolic compounds, genes controlled by P_vanABK_^*^ were efficiently turned off, allowing the control of gene expression in *C. glutamicum* in a timed manner.

Finally, a substantial part of this article Research Topic reports bioproduction of chemicals and proteins by *C. glutamicum*. Sato et al. engineered a *C. glutamicum* strain with β-glucosidase secreting ability for production of shikimate from both glucose and cellobiose. The shikimate pathway is a common route for the biosynthesis of a range of aromatic compounds, which also provides precursors for the biosynthesis of coenzyme Q10, an electron carrier in aerobic respiration and an antioxidant in medical treatment. Burgardt et al. metabolically engineered *C. glutamicum* for *de novo* biosynthesis of coenzyme Q10, which to the best of our knowledge is the first report of coenzyme Q10 production in a non-ubiquinone-containing bacterium. Uridine diphosphate-*N*-acetylglucosamine (UDP-GlcNAc) is an acetylated amino sugar nucleotide that can serve as a sugar donor for synthesis of many pharmaceutically relevant oligosaccharides, polysaccharides, and glycoproteins. Gauttam et al. constructed a series of recombinant *C. glutamicum* strains by engineering the UDP-GlcNAc biosynthetic pathway and the highest level of microbial production of UDP-GlcNAc was obtained. Different from most metabolic engineering strategies directly targeting the biosynthetic pathway, Du et al. applied indirect metabolic engineering strategies targeting the substrate uptake, membrane composition, oxygen transfer, and nitrogen metabolism for enhanced biosynthesis of hyaluronic acid. Metabolic engineering approaches have also been combined with process engineering approaches by Schwentner et al. to develop a *C. glutamicum* strain for the production of compatible solute mannosylglycerate and an easy product separation process to extract mannosylglycerate from cytosol by cold water shock.

In addition to bioproduction of chemicals, secretory production of recombinant proteins using *C. glutamicum* also attracts great attention. Lee et al. found that mutations to increase the iron and carbon consumption were responsible for the enhanced production of recombinant protein in an evolved fast-growing *C. glutamicum* strain. Balasubramanian et al. screened *C. glutamicum* mutants with enhanced enzyme secretion capacity by using a droplet-based microfluidic high-throughput screening method and analyzed the single nucleotide variants in these mutants. Bakkes et al. used a fluorescence-based biosensor for Sec-dependent protein secretion to evolve the Sec signal peptides from *Bacillus subtilis* and optimize the secretion of heterologous enzyme cutinase. These findings are expected to promote the application of *C. glutamicum* in protein production.

Overall, *C. glutamicum,* that was initially used as an l-glutamate producer, is now becoming a versatile chassis for bioproduction of various amino acids, chemicals, and proteins from renewable feedstocks. The importance of *C. glutamicum* in industrial biotechnology drives the development of advanced synthetic and systems biology technologies such as *in silico* metabolic modeling, multiplex gene editing, multi-omics analyses, and high-throughput functional genomics methods. It is believed that the series of articles collected in this Research Topic provide contemporary and valuable information that can help to fully unlock the potential of *C. glutamicum* in industrial biotechnology.
